# Childhood Health and Educational outcomes afteR perinatal Brain injury (CHERuB): protocol for a population-matched cohort study

**DOI:** 10.1136/bmjopen-2024-089510

**Published:** 2024-08-19

**Authors:** Philippa Rees, Chris Gale, Cheryl Battersby, Carrie Williams, Mitana Purkayastha, Ania Zylbersztejn, Ben Carter, Alastair Sutcliffe

**Affiliations:** 1 Population Policy and Practice, University College London Institute of Child Health, London, UK; 2 Neonatal Medicine, School of Public Health, Imperial College London, London, UK; 3 Neonatal Medicine, Imperial College London, London, UK; 4 Biostatistics & Health Informatics, King’s College London, London, UK

**Keywords:** EPIDEMIOLOGIC STUDIES, NEONATOLOGY, PAEDIATRICS

## Abstract

**Abstract:**

**Introduction:**

Over 3000 infants suffer a brain injury around the time of birth every year in England. Although these injuries can have important implications for children and their families, our understanding of how these injuries affect children’s lives is limited.

**Methods and analysis:**

The aim of the CHERuB study (Childhood Health and Educational outcomes afteR perinatal Brain injury) is to investigate longitudinal childhood health and educational outcomes after perinatal brain injury through the creation of a population-matched cohort study. This study will use the Department of Health and Social Care definition of perinatal brain injury which includes infants with intracranial haemorrhage, preterm white matter injury, hypoxic ischaemic encephalopathy, perinatal stroke, central nervous system infections, seizures and kernicterus. All children born with a perinatal brain injury in England between 2008 and 2019 will be included (n=54 176) and two matched comparator groups of infants without brain injury will be created: a preterm control group identified from the National Neonatal Research Data Set and a term/late preterm control group identified using birth records. The national health, education and social care records of these infants will be linked to ascertain their longitudinal childhood outcomes between 2008 and 2023. This cohort will include approximately 170 000 children. The associations between perinatal brain injuries and survival without neurosensory impairment, neurodevelopmental impairments, chronic health conditions and mental health conditions throughout childhood will be examined using regression methods and time-to-event analyses.

**Ethics and dissemination:**

This study has West London Research Ethics Committee and Confidential Advisory Group approval (20/LO/1023 and 22/CAG/0068 issued 20/10/2022). Findings will be published in open-access journals and publicised via the CHERuB study website, social media accounts and our charity partners.

**Strengths and limitations of this study**

- The CHERuB (Childhood Health and Educational outcomes after perinatal Brain injury) study is a population birth cohort study of all children with perinatal brain injuries born in England including those with intracranial haemorrhage, preterm white matter injury, hypoxic ischaemic encephalopathy, perinatal stroke, central nervous system infections, seizures and kernicterus.
- Comparator children have been matched to increase precision and power to detect differences in developmental outcomes between those with and without brain injury above and beyond the impact of factors such as prematurity.
- The longitudinal nature of the study enables the examination of important outcomes across the health, education and social sectors after perinatal brain injury up to 13 years of age.
- This study involves the creation of a rolling birth cohort permitting on-going follow-up of this cohort and the addition of future epochs.
- A key weakness of using routine data is that certain outcome conditions may be under-represented—although this is offset by the triangulation of multiple data sets.

## Introduction

 Perinatal brain injuries can have devastating consequences for children, families and society.^1^ As such, reducing the number of infants with perinatal brain injury is a current governmental priority. Over 3000 infants suffer a perinatal brain injury in England every year and the Department of Health and Social Care (DHSC) has declared a national ambition to halve the rate of perinatal brain injury by 2030.^1 2^

As part of this national maternity ambition, the DHSC commissioned the Neonatal Data Analysis Unit to develop a standardised definition of perinatal brain injury through expert consensus.^1^ This definition includes: moderate-to-severe hypoxic ischaemic encephalopathy (HIE), perinatal stroke, central nervous system infections, intracranial haemorrhage, cystic periventricular leukomalacia, kernicterus and seizures in term and preterm babies; conditions are not mutually exclusive. By its nature, this definition of perinatal brain injury comprises markers of potential brain injury rather than confirmed brain injury, as such injuries are difficult to definitively diagnose during the neonatal period and require long-term follow-up to determine consequent impacts of the perinatal insult. Current knowledge about childhood trajectories after perinatal brain injury is limited by a paucity of population-level research.^3 4^

Most neonatal studies follow-up infants to 2 years of age and measure a composite outcome of mortality and neurodevelopmental impairment. It is however becoming increasingly clear that 2-year outcome measures are poorly predictive of future childhood function, except among the most severely impaired.^5 6^ The poor predictive value of neurodevelopmental testing at 2 years of age is thought to be underpinned by measurement error, difficulties in infant testing (ie, observing a child at one time point), changes in function throughout childhood, variation in inherited developmental patterns and environmental influences.^7^,^9^ Additionally, functional impairment as a result of neonatal insults is fluid: it may evolve or even diminish throughout childhood as a result of neuroplasticity. A greater understanding of the sequelae of perinatal brain injury, specifically how and when children are affected, would inform parental counselling, enhanced developmental surveillance across the National Health Service (NHS) and the design of multidisciplinary interventions to support children in reaching their full potential.^10^

### Aim

The CHERuB (Childhood Health and Educational outcomes after perinatal Brain injury) study is a population birth cohort study that aims to investigate longitudinal childhood health and educational outcomes after perinatal brain injury. We propose using a national database capturing all children with perinatal brain injuries admitted to a neonatal unit and two matched control cohorts, linked to administrative health, education and social care data to ascertain long-term outcomes.

### Objectives

- Work with affected families to inform the study design, focus, interpretation of results and their dissemination.
- Create a population-based national matched cohort of children with and without perinatal brain injury from existing national data sets.
- Examine neurodevelopmental, school and survival outcomes after perinatal brain injury up to 13 years of age compared with unaffected peers.
- Examine the prevalence and nature of chronic health conditions in children with perinatal brain injury compared with unaffected peers.
- Examine mental health outcomes after perinatal brain injury compared with unaffected peers.

## Methods and analysis

### Study population

The CHERuB study will include approximately 170 996 children: all infants born in England between 1 January 2008 and 31 December 2019 who were admitted to a neonatal unit with a perinatal brain injury (cohort 1; n=54 176) and two matched comparator groups of infants admitted to the neonatal unit and born at <34 (cohort 2) and infants born at ≥34 weeks’ gestation (cohort 3).

Cohort 1 will include infants with perinatal brain injury as per the DHSC definition in addition to infants with intraventricular haemorrhage grades 1–2 and mild HIE (online supplemental file 1).^1^ Infants born outside of England or with congenital infections, encephalopathies or brain abnormalities will not be included.^1^

### Identifying comparators

Infants with perinatal brain injury born at <34 weeks’ gestation will be matched 1:1 with unaffected infants admitted to a neonatal unit using propensity scores accounting for the week of gestation, birth weight Z-score, sex, mode of delivery, multiplicity, maternal smoking status, receipt of antenatal steroids, receipt of antenatal magnesium sulfate and year of birth (cohort 2). This will be done using Mahalanobis matching (in the psmatch2 function in Stata) to identify the 20 nearest neighbour matches for each preterm infant with brain injury based on the prespecified covariates.^11^ The nearest unique match (of the 20) will then be selected to minimise duplicate comparators. Additional confounders affecting the preterm population necessitate more complex matching to create a balanced comparable cohort for meaningful analysis.

Infants with perinatal brain injuries born at 34 weeks’ gestation or greater will be matched to unaffected comparator infants indicated from a database of all births in England in a 1:3 ratio on sex, year of birth, birth weight (within 100 g), week of gestation (from 2015) and multiplicity (cohort 3).

### Follow-up

Included children will be followed-up to 13 years of age, 31 August 2023, or their date of death through linked hospital, death, school and social care records. Follow-up time will vary by birth year.

### Data sources

Cohorts 1 and 2 will be derived from the National Neonatal Research Database (NNRD). The NNRD contains demographic, clinical and organisational data derived from electronic patient records for all neonates admitted to a neonatal unit across Great Britain.^12 13^ The NNRD also captures information on short-term developmental outcomes at 2 years of corrected age for certain groups of neonates.^13^ Cohort 3 will be derived from Office for National Statistics birth registration data which pertains to all births registered in England. These data sets are described in brief in table 1.

**Table 1:** Summary of proposed data sets

Data sources	Description of data set
**National Neonatal Research Database(NNRD)**	Overseen by the Neonatal Data Analysis Unit and contains demographic, clinical and administrative data for all neonates admitted to neonatal units across Great Britain.^13^ Its population coverage is internationally unique with 100% coverage of England and Wales since 2012 and high representative coverage since 2008.^12^ It also contains 2-year neurodevelopmental follow-up records for certain high-risk groups of neonates.^13 27^
**Personal Demographic Service (PDS**)	Controlled by NHS England, the PDS contains demographic data for all NHS patients in England with an NHS number.
**Hospital Episode Statistics (HES)**HES admitted patient careHES accident and emergencyHES emergency care data setHES outpatientsHES-ONS linked mortality data	Controlled by NHS England. Contains all data pertaining to NHS-funded hospital admissions, outpatient appointments and emergency department attendances across England.^28^ It has been extensively used to investigate longitudinal population health outcomes because it is uniquely positioned to do so with its universal coverage and patient-level data.^28 29^Additionally, ONS mortality records are routinely linked to HES records to provide population data on cause and time of death.
**Mental Health Services Data Set**	Controlled by NHS England. Contains individual-level data for all children accessing mental healthcare across the community, outpatient and inpatient settings in England from 2016.
**National Pupil Database (NPD)**School enrolment:NPD school census pupil levelNPD pupil referral unit censusNPD alternative provision censusAttainment:NPD early years foundation stage profileNPD phonicsNPD key stage 1NPD key stage 2Other:NPD absencesNPD exclusions	Controlled by the Department for Education. Contains detailed pupil-level information on characteristics of pupils in state-funded schools, their educational attainment, special educational needs provision and attendance of all children at state schools across England. These data have previously been linked to health data successfully with linkage rates of over 80%.^15 30^
**Social care data**Children Looked After Return (CLA)Child In Need Census (CIN)	The CLA is a national data set that contains data on all looked-after children in England.^31^ It is held by the Department for Education and contains individual-level data.The CIN is also a national individual-level data set held by the Department for Education.^26^ It contains longitudinal data on children deemed to be in need, that is, those who have been referred to the local authority for social care support in England. Referrals accepted by the local authority are included in the CIN. These include children in care, children on child protection plans and disabled children (who are receiving services from the local authority). Looked-after children appear in both the CLA and CIN data sets.

NHSNational Health ServiceONSOffice for National Statistics

Follow-up data will be obtained from the NNRD and linkage to administrative health and education records. All data sets are described in table 1. In brief, we will use:

- Diagnostic data recorded during hospital presentations, admissions and appointments in health records to measure indicators of neurodevelopmental impairments, chronic conditions and mental health conditions.
- ONS-Hospital Episode Statistics (HES) linked mortality records to obtain information about causes and timing of death.
- Education records to obtain information about educational attainment, level of provision and recorded type of special educational needs.

### Data linkage

**Figure 1:**
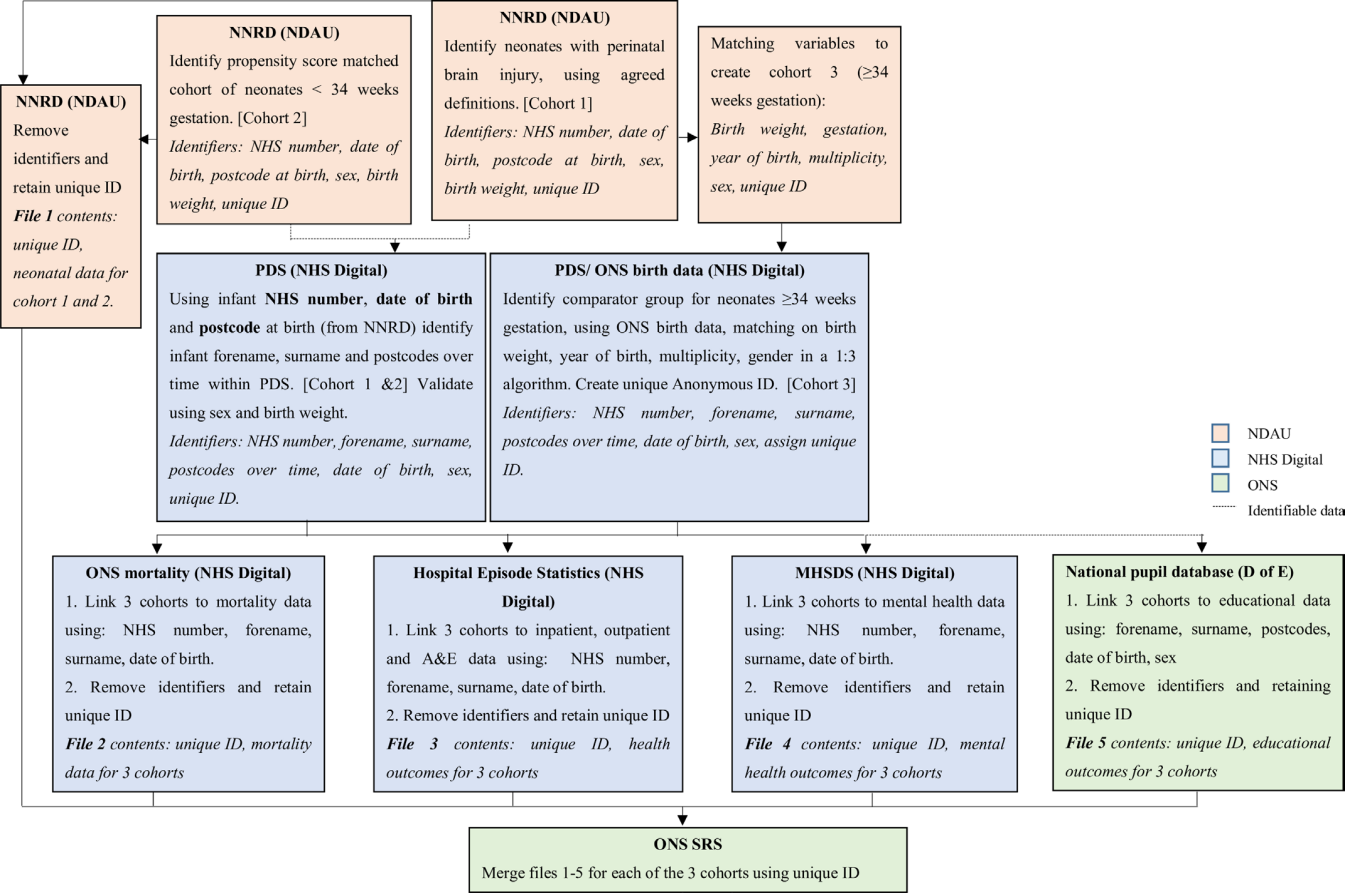
CHERuB study data flows. MHSDS, Mental Health Services Data Set; NDAU, Neonatal Data Analysis Unit; NHS, National Health Service; NNRD, National Neonatal Research Database; ONS, Office for National Statistics; PDS, Personal Demographic Service.

The suggested data flows for study data are outlined in figure 1. The main steps are:

1. Extraction and transfer of NNRD data to the ONS Secure Research Service (SRS): Infants with perinatal brain injury will be identified within the NNRD (cohort 1; n=54 176). The pseudonymised neonatal care data for this cohort will be transferred to the ONS SRS. Preterm infants (<34 weeks’ gestation) will be propensity score matched to a comparator group of infants in the NNRD (cohort 2; n=22 854). The pseudonymised neonatal care data for cohort two will also be transferred to the ONS SRS.
2. Linkage of the NNRD to the Personal Demographic Service (PDS): The NNRD reliably captures data items such as date of birth, postcode and infant NHS number. The NNRD cohorts will be linked to the PDS, a database that contains demographic data for all NHS patients in England with an NHS number and serves as a linkage spine for health records. Linkage will be done using NHS number, date of birth, sex and postcode at birth: to identify registered forename, surname and postcodes changes (which are required for linkage to education records).
3. Deriving cohort 3: The remaining unmatched infants (≥34 weeks’ gestation) with perinatal brain injury will be matched in a 1:3 ratio to a comparator group of infants, identified from ONS birth notifications by NHS England (cohort 3; n=93 966).
4. Linkage to health data sets via the PDS: Using NHS number, infant surname, forename, sex and date of birth, all three cohorts will be identified in the PDS by NHS England using their Master Person Service bespoke deterministic person-matching algorithm.^14^ The PDS serves as a spine for linkage to routinely collected health records: ONS mortality records, HES and the Mental Health Services Data Set (MHSDS). The data will be linked to the most recent available health records for the included children up to 13 years of age.
5. Linking to education and social care data: One file containing a list of personal identifiers for the linkage of the three cohorts to the education and social care data sets will be transferred to the Department for Education. The following identifiers will be used by the Department for Education to link to education and social care data: forename, surname, date of birth, sex and postcodes.^15^ Another file, containing the three pseudonymised cohorts of infants and their health outcomes, will be transferred to the ONS SRS.

### Outcomes of interest

1. Survival and neurodevelopmental impairment.

The primary outcome of interest, survival without neurosensory impairment, will be determined up to 2, 5, 7 and 11 years of age using ONS mortality, HES, NNRD and National Pupil Database (NPD) records as binary outcomes at four time points. Two-year outcomes will be presented corrected and uncorrected for prematurity where possible.

The specific definitions for neurosensory impairment are denoted in online supplemental file 2 and include cognitive, motor, speech and language, visual and hearing impairments. The potential of extending this definition to include data from the MHSDS will be explored on data receipt as the utility of the MHSDS (available from 2016) for paediatric studies is still uncertain.

Survival without neurosensory impairment is a priority outcome for parents (as highlighted by previous core outcome studies in neonatology and HIE specifically).^16 17^ This definition of survival without neurosensory impairment has been developed by scoping the existing literature and through expert consensus with patient and public involvement (PPI) representatives, community paediatricians, general paediatricians, neonatologists and academics with expertise in NNRD, HES and NPD data.

We will consider the individual components of the composite primary outcome in secondary analyses as well as other important neurodevelopmental outcomes (onlinesupplemental files 2^ 3^). These include:

- Neurosensory impairment (cognitive, motor, speech and language, hearing or visual).
- Any neurodevelopmental impairment.
- Cognitive impairment.
- Not meeting expected level of academic attainment.
- Learning difficulty components of special educational needs.
- Motor/physical impairment.
- Speech, language and communication difficulty.
- Visual impairment.
- Hearing impairment.
- Additional educational needs (any recorded special educational needs, Educational Health and Care Plan and attending a specialist school).
- Developmental delay (although we recognise this is not an impairment).
- Epilepsy.
- Behavioural, emotional and social disorders (hyperkinetic, autism spectrum disorder, tic disorder).
- Survival.
- Cause-specific mortality.

2. Chronic health conditions (* outcomes are not mutually exclusive; some conditions are classed as both a type of neurodevelopmental impairment and a chronic health condition).

This is defined as the occurrence of the chronic health conditions included in the Hardelid classification up to age 2, 5 and 11 years.^18^

- Infections.
- Cancer and chronic blood conditions.
- Cardiovascular conditions.
- Respiratory conditions.
- Musculoskeletal/dermatological conditions.
- Neurological conditions.
- Metabolic/endocrine/digestive conditions.
- Renal/genitourinary conditions.

3. Mental health conditions (* outcomes are not mutually exclusive; some conditions are classed as both an NDI and a mental health condition).

This is defined as the occurrence of behavioural, emotional, pervasive, mood and/or anxiety disorders up to age 5 and 11 years of age in HES or NPD data (online supplemental file 4). The potential of extending this definition to the MHSDS will be explored on data receipt.

### Potential confounding or effect modifiers

Directed acyclic graphs (DAGs) will be created to map the relationships of the variables in table 2, types of exposures (ie, brain injuries) and outcome types.^19^ Selection of key covariates for each analysis will be guided ‘a priori’ by the DAGs. Potential effect modifiers will also be identified using DAGs and explored further within each analysis through stratification.

**Table 2:** Potential confounders or effect modifiers within the linked data sets available for analysis in addition to the outcome variables (*matched variable)

NNRD (cohort 1 and 2 only)	PDS/HES	NPD and social care data
Year of birth*Month/season of birthSex*Gestation*Birth weight Z-score*Multiplicity*Antenatal corticosteroids*Antenatal magnesium sulfate*Receiving breastmilk at dischargeMode of delivery**Neonatal unit characteristics**Admission neonatal unit levelAdmitting operational delivery networkDischarging neonatal unit levelDischarging operational delivery networkNumber of different neonatal unit admissions**Maternal characteristics**Maternal smoking statusIndex of multiple deprivationMaternal ethnicityMaternal age**Neonatal multimorbidity**Necrotising enterocolitis (conservatively or surgically treated)Spontaneous intestinal perforationRetinopathy of prematurity (treated)Bronchopulmonary dysplasiaCulture positive sepsisMajor surgeryPatent ductus arteriosus (treated)More than one brain injury**Treatment variables**Duration of ventilationReceipt of surfactantPostnatal corticosteroid treatmentFeeding regimes (feeding routes, types and durations)Therapeutic hypothermia (for infants with HIE)	Season of birthYear of birth*Sex*Gestation*Birth weight*Multiplicity*Maternal ethnicityChild ethnicityIndex of multiple deprivationHospital of birth	School attendance (and exclusions)Child in needLooked after childEligibility for free school mealsFirst languageChild ethnicityQuintile of index of multiple deprivation

HESHospital Episode StatisticsHIEHypoxic Ischaemic EncephalopathyNNRDNational Neonatal Research DatabaseNPDNational Pupil DatabasePDSPersonal Demographic Service

### Statistical analysis

Analyses will be reported as per the Reporting of studies conducted using observational routinely-collected health data (RECORD) guidelines.^20^

The probability of survival without neurosensory impairment among the included population up to 2, 5, 7 and 11 years of age will be determined using multilevel logistic regression methods adjusting for the key covariates identified from DAGs and fitting hospital of birth as a random effect (to account for heterogeneity at the hospital level). The crude and adjusted ORs will also be calculated and presented for each of the secondary outcomes (ie, type of neurodevelopment diagnosis or additional needs) and by brain injury type accounting for the competing risk of death. These methods will also be used to study the risk of chronic health conditions and mental health conditions after perinatal brain injury. We will use Stata V.18 (or later) software for all analyses.

Further analysis of academic attainment will be undertaken using a linear regression model (adjusting for the key covariates) to determine the differences in mean academic attainment Z-scores on both teacher and national assessments at Phonics, Early Years Foundation Stage (EYFS), Key Stage 1 (KS1) and Key Stage 2 (KS2) after perinatal brain injury and by type of brain injury. This will also be undertaken for the specific domains that make up the overall teacher and/or national scores at each of the educational assessment stages (table 3).

**Table 3:** Educational assessments and domains

Educational assessment stages	Domains assessed
Phonics	Phonics assessment (teacher-marked assessment out of 40)
Early Years Foundation Stage(all teacher-assessed)	Achieved good level of development overall (binary)Subcategories (categorical score between 1 and 3) Communication. Physical development. Personal, social and emotional development. Literacy. Mathematics. Understanding the world. Expressive arts, designing and making.
Key Stage 1	English (national assessment and teacher-assessed). Mathematics (national assessment and teacher-assessed). Science (teacher-assessed).
Key Stage 2	English reading (national assessment). English grammar (national assessment). English punctuation (national assessment). English spelling (national assessment). English writing (teacher-assessed). Mathematics (national assessment). Science (teacher-assessed).

Data permitting, a group trajectory analysis may be undertaken to study the differing academic trajectories at EYFP, KS1 and KS2 of infants with perinatal brain injury and to determine if and how these differ by brain injury type.^21 22^ This could provide insights into whether impairments as a result of specific perinatal brain injuries are fixed, deteriorate or even improve throughout childhood, in addition to identifying characteristics associated with these different trajectories.

To determine the difference in absolute mortality rates (and 95% CI) per 1000 person-years between those with and without perinatal brain injury (and by brain injury type) a survival analysis with Cox proportional hazards modelling will be undertaken. This will include censoring follow-up time, fitting the admission hospital or neonatal operational delivery network (data permitting) as a random effect using the frailty function, adjusting for covariates identified from DAGs and examining the data for breaches in the proportionality assumption.

### Subgroup analyses

The primary and secondary outcomes will be reported for those with and without brain injury but also by type of brain injury. Additionally, outcomes for these specific populations will be explored geographically, temporally and in relation to specific treatments. Additional subgroup analyses of neurodevelopmental and school-based outcomes will include exploring how these outcomes differ based on eligibility for free school meals, being a ‘looked after child’, first language spoken at home, exclusions from schools and school type.

### Missing outcome data

Infants from cohorts 1 and 2 (identified from the NNRD) with and without a 2-year follow-up record in the NNRD will be compared. Sensitivity analyses will be undertaken to assess the potential impact of missing data. For cohorts 1 and 2 the standardised difference for those with and without 2-year follow-up data in the NNRD will be calculated for the NNRD covariates specified in table 1. The standardised difference for those with and without 2-year follow-up data will also be calculated by the type of perinatal brain injury for cohort 1. The predominant mechanism of missing 2-year NNRD follow-up data, is likely to be missing at random (MAR), that is, predictable missingness related to covariates such as ethnicity, neonatal network responsible for follow-up and index of median deprivation.^23^ Some neonatal follow-up studies have shown an element of missing not at random (MNAR) in their missing follow-up data, where children with NDI are less likely to attend research study follow-up (due to a myriad of factors such as the burden of care appointments independent of ‘research’ appointments).^23^,^25^ Other studies have demonstrated that children without NDI might be less likely to attend follow-up.^26^ However, these were typically follow-up studies conducted alongside, rather than as part of, routine care. The data in the CHERuB study are collected as part of routine care, we would therefore expect this MNAR missingness mechanism to play less of a role. To explore the potential impact of MNAR, sensitivity analyses will be undertaken to simulate violations of the MAR assumption in either direction. The use of inverse probability weighting or multiple imputation methods will be considered to adjust for sources of potential bias resulting from missing outcome data.

The incidence of specific NDIs, chronic health conditions and mental health conditions in cohort 3 will be compared with estimates of the prevalence of these conditions within the general UK population from the literature and existing registries to assess the validity of the CHERuB study estimates.

### Examining linkage quality

The linkage rate for cohort 1 and 2 from the NNRD to a HES records, and for cohort 3 from the ONS birth notifications to a HES records will be calculated. The characteristics of infants in cohorts 1–3 who did and did not link to a HES record will be compared by calculating standardised differences for the year of birth, sex, maternal ethnicity and index of multiple deprivation decile for each group, to evaluate for potential linkage bias as a result of missed matches.^15^ Furthermore a multivariate logistic regression analysis will be undertaken to determine whether these variables independently increase the risk of linkage failure. If necessary, multiple imputation methods will be considered to adjust for potential sources of bias resulting from linkage error.

The linkage rates of cohorts 1–3 from HES to the NPD will be calculated; and the characteristics of infants from the NNRD who did and did not match to an NPD record in addition to those from cohort 3 in HES who did and did not match to an NPD record, will be compared using the same methods as for the NNRD-HES linkage evaluation.

### Patient and public involvement

The study and in particular the PPI components of the study are overseen by a PPI advisory committee which consists of parent representatives and representatives from Bliss, Peeps HIE and the meningitis research foundation. Families of infants with perinatal brain injury have also been recruited via charity partners (Bliss and Peeps HIE) to participate in PPI workshops. Early family workshops have been undertaken to inform the study design and focus. Further workshops are planned later in the study to explore the study results with families: particularly what specific results might mean to families and to seek their input on how best to disseminate and use these findings going forward.

## Ethics and dissemination

The study has received West London Research Ethics Committee (20/LO/1023) and Confidential Advisory Group approval (22/CAG/0068).

Pseudonymised data will be stored in the ONS SRS and only accessible to specific ONS accredited researchers working on this study. No identifiable data are made available to researchers at any time. NHS England and the Department for Education will retain the linkage keys for their respective linkages, to permit future linkage updates.

Receipt of the full linked cohort is anticipated by September 2024. We aim to complete and disseminate the study findings by the study end date in August 2026. A cohort description will be published in a peer-reviewed journal. Study findings will be published open-access in high-impact journals targeting key stakeholders and presented at national and international conferences. The public and families will be signposted to study summaries and publications via the CHERuB study website https://www.ucl.ac.uk/child-health/research/ppp/champp/cherub, the study social media account @CHERuBstudy and our charity partners.

## supplementary material

10.1136/bmjopen-2024-089510 online supplemental file 1

10.1136/bmjopen-2024-089510 online supplemental file 2

10.1136/bmjopen-2024-089510 online supplemental file 3

10.1136/bmjopen-2024-089510 online supplemental file 4
