# Supplementary material for: Childhood Health and Educational outcomes afteR perinatal Brain injury (CHERuB): protocol for a population-matched cohort study
Source: BMJ Open. 2024 Aug 19;14(8):e089510. doi: 10.1136/bmjopen-2024-089510 (PMC11337658; doi:10.1136/bmjopen-2024-089510)
Supplement: online supplemental file 1 [file bmjopen-14-8-s001.pdf]

Supplement 1: Definition of perinatal brain injury used in the CHERuB study

Extensions to the Department of Health and Social Care definition:

Any of the following recorded in any *Diagnosis* field:

- Grade 1 Hypoxic Ischaemic Encephalopathy (HIE)
- Mild Neonatal Encephalopathy
- Mild Hypoxic Ischaemic Encephalopathy HIE)

Any of the following recorded in any *Diagnosis* field:

1. Intraventricular haemorrhage (IVH Grade 1)
2. Intraventricular haemorrhage (IVH Grade 2)

Any of the following recorded in any *cranial ultrasound findings* field:

1. IVH Grade 1
2. IVH Grade 2

Department of Health and Social Care definition of brain injury:

HIE

Any of the following recorded in any *Diagnosis* field:

1. Severe Hypoxic Ischaemic Encephalopathy (HIE)
2. Severe Neonatal Encephalopathy
3. Grade 3 Hypoxic Ischaemic Encephalopathy (HIE)
4. Moderate Hypoxic Ischaemic Encephalopathy (HIE)
5. Moderate Neonatal Encephalopathy
6. Grade 2 Hypoxic Ischaemic Encephalopathy (HIE)

OR

The following recorded in *daily care neurology* field:
Therapeutic hypothermia induced - for 2 or more consecutive days

Seizures

Any of the following recorded in any *daily care neurology* field:
1. Seizure occurred

Intracranial haemorrhage

Any of the following recorded in any *Diagnosis* field:

3. Subdural haemorrhage due to birth injury
4. Cerebral haemorrhage due to birth injury
5. Traumatic intraventricular haemorrhage
6. Subarachnoid haemorrhage due to birth injury
7. Subarachnoid haemorrhage
8. Tentorial tear due to birth injury
9. Intracranial laceration and haemorrhage due to birth injury
10. Large intraventricular haemorrhage (IVH Grade 3)
11. Intraventricular haemorrhage/parenchymal
12. Parenchymal haemorrhage
13. Intraventricular haemorrhage (IVH Grade 4)
14. Intracranial Haemorrhage (unknown or unspecified cause)
15. Intracerebral haemorrhage
16. Intracerebral haemorrhage (term infant)
17. Intraventricular haemorrhage (perinatal)
18. Post-haemorrhagic hydrocephalus

OR

Any of the following recorded in any *cranial ultrasound findings* field:

3. Large intraventricular haemorrhage (IVH Grade 3)
4. Intraventricular haemorrhage/parenchymal haemorrhage (IVH Grade 4)
5. Parenchymal haemorrhage

OR

Any of the following recorded in any *procedure field*:
1. Ventriculoperitoneal or other ventricular shunt
2. External ventricular drain
3. Ventricular drain with reservoir
4. Insertion of ventricular peritoneal shunt.
5. Insertion of Rickham reservoir
6. Insertion of ventriculo-atrial CSF shunt
7. Insertion of ventriculo-peritoneal CSF shunt

Creation of ventriculoperitoneal shunt

Preterm white matter injury

Any of the following recorded in any *Diagnosis* field:
1. Cystic periventricular leukomalacia

OR

Any of the following recorded in any *cranial ultrasound findings* field:
Cystic periventricular leukomalacia

Perinatal stroke

Any of the following recorded in any *Diagnosis at discharge* field:
1. Neonatal stroke
2. Infarction: Middle cerebral artery (stroke)
3. Cerebrovascular accident (stroke)
4. Cerebral venous thrombosis

Neonatal cerebral ischaemia

Central Nervous System Infection

Any of the following diagnosis codes recorded in the *diagnosis* field:
1. Bacterial meningitis
2. Viral meningitis
3. Meningitis – streptococcal
4. Meningitis – bacterial (specific organism)
5. Meningitis – bacterial (unknown or unspecified organism)
6. Meningitis – Candida
7. Candida encephalitis
8. Congenital herpes infection

Any pathogen recorded in the *suspected infection data* field *Pathogen in CSF*

Kernicterus

Any of the following diagnoses recorded in any *Diagnosis* field:
1. Bilirubin encephalopathy (immune)
2. Kernicterus (unspecified or unknown cause)
