# Supplementary material for: Childhood Health and Educational outcomes afteR perinatal Brain injury (CHERuB): protocol for a population-matched cohort study
Source: BMJ Open. 2024 Aug 19;14(8):e089510. doi: 10.1136/bmjopen-2024-089510 (PMC11337658; doi:10.1136/bmjopen-2024-089510)
Supplement: online supplemental file 2 [file bmjopen-14-8-s002.pdf]

Supplement 2: The CHERuB study definition of survival without neurosensory impairment

Data source	Domains/ conditions	Definition
NNRD	Developmental delay	• Mild developmental delay (3-6 months)<br>• Moderate developmental delay (6-12 months)<br>• Severe developmental delay (>12 months)
	Communication	• Any difficulty with communication<br>• Difficulty with speech (<10 words or signs)<br>• Difficulty understanding outside of familiar context<br>• Has <5 meaningful words, vocalisations, or signs<br>• Unable to understand words or signs
	Motor	• Any difficulty walking<br>• Unable to walk without assistance<br>• Non-fluent or abnormal gait reducing mobility<br>• Difficulty with the use of both hands<br>• Difficulty with the use of one hand<br>• Unable or needs support sitting<br>• Unable to sit<br>• Unable to use hands e.g. to feed
	Vision	• Any vision problems including squint<br>• A not fully correctable visual defect<br>• Complete blindness or able to see light only
	Auditory	• A hearing impairment<br>• Hearing impairment correctable with aids<br>• Hearing impairment not correctable with aids
HES and ONS Mortality Records	Survival	• The absence of a death record in a linked HES record
	Cognitive impairment	F70-79 Intellectual impairment<br>F81.0 Specific reading disorder<br>F81.1 Specific spelling disorder<br>F81.2 Specific disorder of arithmetical skills<br>F81.3 Mixed disorder of scholastic skills<br>F81.8 Other developmental disorders of scholastic skills<br>F81.9 Developmental disorder of scholastic skills, unspecified<br>R48.0 Dyslexia and alexia<br>R 48.1 Agnosia<br>R48.2 Apraxia<br><br>R48.8 Other and unspecified symbolic dysfunctions
	Developmental disorders of speech and language	F80.0 Specific speech articulation disorder<br>F80.1 Expressive language disorder<br>F80.2 Receptive language disorder<br>F80.3 Acquired aphasia with epilepsy [Landau-Kleffner]

		F80.9 Developmental disorder of speech and language, unspecified<br>R47.0 Dysphasia and aphasia<br>R47.1 Dysarthria and anarthria<br>R47.8 Other and unspecified speech disturbances
	Specific developmental disorder of motor function/ dystonia/ cerebral palsy	F82 Specific developmental disorder of motor function<br>G24.9 Dystonia, unspecified<br>G80 Cerebral palsy<br>G81 Hemiplegia<br>G82 Paraplegia and tetraplegia<br>G83.0 Diplegia of upper limbs<br>G83.1 Monoplegia of lower limb<br>G83.2 Monoplegia of upper limb<br>G83.4 Cauda equina syndrome<br>G83.5 Locked in syndrome<br>G83.6 Upper motor neuron facial paralysis<br>R26.0 Ataxic gait<br>R26.1 Paralytic gait<br>R27.0 Ataxia unspecified<br>R27.8 Other and unspecified lack of coordination<br>G25.9 Extrapyramidal and movement disorder, unspecified
	Vision	H47.6 Disorders of visual cortex<br>H51.0 Palsy of conjugate gaze<br>H51.1 Convergence insufficiency and excess<br>H51.2 Internuclear ophthalmoplegia<br>H51.8 Other specified disorders of binocular movement<br>H51.9 Disorder of binocular movement, unspecified<br>H52.0 Hypermetropia<br>H52.1 Myopia<br>H52.2 Astigmatism<br>H52.3 Anisometropia and aniseikonia<br>H52.4 Presbyopia<br>H52.5 Disorders of accommodation<br>H52.6 Other disorders of refraction<br>H52.7 Disorder of refraction, unspecified<br>H 53.0 Amblyopia ex anopsia<br>H53.4 Visual field defects<br>H55 Nystagmus and other irregular eye movements<br><br>H54.0 Blindness, binocular<br>H54.1 Severe visual impairment, binocular<br>H54.2 Moderate visual impairment, binocular<br>H54.4 Blindness, monocular<br>H54.5 Severe visual impairment, monocular<br>H54.6 Moderate visual impairment, monocular<br>H54.9 Unspecified visual impairment (binocular)

	Hearing	H90.3 Sensorineural hearing loss, bilateral<br>H90.4 Sensorineural hearing loss, unilateral with unrestricted hearing on the contralateral side<br>H90.5 Sensorineural hearing loss, unspecified<br>H90.6 Mixed conductive and sensorineural hearing loss, bilateral<br>H90.7 Mixed conductive and sensorineural hearing loss, unilateral with unrestricted hearing on the contralateral side<br>H90.8 Mixed conductive and sensorineural hearing loss, unspecified<br><br>D24 Operations on cochlea<br>D24.1 Implantation of intracochlear prosthesis<br>D24.2 Implantation of extracochlear prosthesis<br>D24.3 Attention to cochlear prosthesis<br>D24.4 Neurectomy of cochlea<br>D24.6 Removal of cochlear prosthesis<br>D24.8 Other specified operations on cochlea<br>D24.9 Unspecified operations on cochlea<br>D13 Attachment of Bone Anchored Hearing Prosthesis<br>D16 Reconstruction of Ossicular Chain<br>D05 Attachment of auricular prosthesis<br>Z453 Adjustment and management of implanted hearing device<br>Z461 Fitting and adjustment of hearing aid<br>Z962 Presence of otological and audiological implants<br>Z974 Presence of external hearing-aid
National Pupil Database	Academic attainment	Did not achieve expected level or the presence of a SEN that precluded assessment at:<br>Early Years Foundation Stage<br>Key Stage 1 national assessment<br>Key Stage 2 national assessment
	Special educational beeds	Indication of type of need for SEN provision (primary or secondary) irrespective of level of SEN provision (SEN support or EHCP)<br><br>- Specific Learning Difficulty<br>- Moderate Learning Difficulty<br>- Severe Learning Difficulty<br>- Profound & Multiple Learning Difficulty<br>- Speech, Language and Communication Needs<br>- Hearing Impairment<br>- Visual Impairment<br>- Multi-Sensory Impairment<br>- Physical Disability
