# Supplementary material for: Childhood Health and Educational outcomes afteR perinatal Brain injury (CHERuB): protocol for a population-matched cohort study
Source: BMJ Open. 2024 Aug 19;14(8):e089510. doi: 10.1136/bmjopen-2024-089510 (PMC11337658; doi:10.1136/bmjopen-2024-089510)
Supplement: online supplemental file 4 [file bmjopen-14-8-s004.pdf]

Supplement 4: The CHERuB study definition of mental health and behavioural conditions		
HES	Behavioural and emotional disorders	F90 Hyperkinetic disorders<br><br>F91 Conduct disorders<br><br>F92 Mixed disorders of conduct and emotions<br><br>F93 Emotional disorders with onset specific to childhood<br><br>F94 Disorders of social functioning with onset specific to childhood and adolescence<br><br>F95 Tic disorders<br><br>F98 Other behavioural and emotional disorders with onset usually occurring in childhood and adolescence
	Pervasive disorders (Autisms)	F84.0 Childhood autism<br><br>F84.1 Atypical autism<br><br>F 84.3 Other childhood disintegrative disorder<br><br>F84.4 Overactive disorder associated with mental retardation and stereotyped movements<br><br>F84.5 Asperger syndrome<br><br>F84.8 Other pervasive developmental disorders<br><br>F84.9 Pervasive developmental disorder, unspecified
	Mood disorders	F30 Manic episode<br><br>F31 Bipolar affective disorder<br><br>F32 Depressive episode<br><br>F33 Recurrent depressive disorder<br><br>F34 Persistent mood [affective] disorders<br><br>F38 Other mood [affective] disorders

		F39 Unspecified mood [affective] disorder
	Anxiety disorders	F40 Phobic anxiety disorders<br><br>F41 Other anxiety disorders<br><br>F42 Obsessive-compulsive disorder<br><br>F43 Reaction to severe stress, and adjustment disorders<br><br>F44 Dissociative [conversion] disorders<br><br>F45 Somatoform disorders<br><br>F48 Other neurotic disorders
NPD	Indication of type of need for special educational need provision (primary or secondary) irrespective of level of provision	Behaviour, emotional & social difficulties (up to 2013/14)
		Social, emotional, and mental health (from 2014/15)
		Autistic spectrum disorder
