# Supplementary material for: Childhood Health and Educational outcomes afteR perinatal Brain injury (CHERuB): protocol for a population-matched cohort study
Source: BMJ Open. 2024 Aug 19;14(8):e089510. doi: 10.1136/bmjopen-2024-089510 (PMC11337658; doi:10.1136/bmjopen-2024-089510)
Supplement: Supplementary file 5 [file bmjopen-2024-089510.reviewer_comments.pdf]

PEER REVIEW HISTORY

BMJ Open publishes all reviews undertaken for accepted manuscripts. Reviewers are asked to complete a checklist review form (**http://bmjopen.bmj.com/site/about/resources/checklist.pdf**) and are provided with free text boxes to elaborate on their assessment. These free text comments are reproduced below.

ARTICLE DETAILS

**TITLE (PROVISIONAL)**	Childhood Health and Educational outcomes afteR perinatal Brain injury (CHERuB): protocol for a population matched cohort study
**AUTHORS**	Rees, Philippa; Gale, Chris; Battersby, Cheryl; Williams, Carrie; Purkayastha, Mitana; Zylbersztejn, Ania; Carter, Ben; Sutcliffe, Alastair

This article was not externally reviewed at BMJ Open. Protocols that have been independently assessed prior to submission to BMJ Open are usually fast-tracked to publication on the grounds that further substantial changes will not be possible. This independent assessment will usually be external, independent review for both a substantial grant award from a non-commercial or government funder and ethics approval.
